# A comparative study of gastric adenocarcinoma HER2 IHC phenotype and mass spectrometry-based quantification

**DOI:** 10.3389/fonc.2023.1152895

**Published:** 2023-06-07

**Authors:** Bin Xu, Hui Chen, Jingjing Zhang, Yanghai Cong, Li Ning, Limin Chen, Yushi Zhang, Yong Zhang, Zhanchun Song, Yuan Meng, Lianqi He, Wei-li Liao, Ying Lu, Fengyi Zhao

**Affiliations:** ^1^ Pathology Department, Fushun Central Hospital, Fushun, Liaoning, China; ^2^ Stomatology Department, Fushun Central Hospital, Fushun, Liaoning, China; ^3^ Technology Department, Tianjin Yunjian Medical Laboratory Co. Ltd., Tianjin, China; ^4^ Medical Oncology, Fushun Central Hospital, Fushun, Liaoning, China; ^5^ Pathology Department, Liaoning Cancer Hospital & Institute, Shenyang, Liaoning, China; ^6^ Circulation Department, Fushun Central Hospital, Fushun, Liaoning, China; ^7^ Research and Development Department, mProbe Inc., Palo Alto, CA, United States; ^8^ Laboratory Medicine, Fushun Central Hospital, Fushun, Liaoning, China

**Keywords:** gastric cancer, mixed classification, HER2, IHC, MS-SRM, types identification

## Abstract

**Introduction:**

Gastric cancer is a highly heterogeneous malignant tumor of the digestive system. Anti-HER2 treatment can inhibit downstream signaling pathways and improve clinical treatment and outcomes in patients with HER2 protein overexpression. Currently, two standard methods for evaluating HER2 expression status are immunohistochemistry (IHC) and fluorescence in situ hybridization (FISH). However, these low-throughput assays often produce discordant or equivocal results.

**Methods:**

In this study, we presented a new HER2 protein detection method based on mass spectrometry selected reaction monitoring (MS-SRM) and validated the method. We conducted a retrospective study on 118 formalin-fixed paraffin-embedded (FFPE) tissues from patients with advanced gastric adenocarcinoma in northern China, and we compared the MS-SRM results with those from IHC and correlated them with FISH.

**Results:**

We established and validated the upper and lower detection limits (300-700 amol/μg) for abnormal HER2 protein expression in advanced gastric cancer. We also found that, among samples with mixed Lauren subtypes, those with a high level of HER2 expression had typical intestinal type features in pathology.

**Discussion:**

This study demonstrated that the MS-SRM method can overcome the limitations and deficiencies of IHC, directly quantify the expression of HER2 protein in tumor cells and be used as a supplement to IHC. It has the potential to be used as a companion diagnosis for new drugs used to treat advanced gastric cancer. Large-scale clinical validation is required.

## Introduction

Gastric cancer is a heterogenous malignant tumor of the digestive system. It is the fifth most common cancer and is the third leading cause of cancer-related death worldwide (1). Because of its high incidence and mortality rate, gastric cancer is an important global health problem (1). China is a high-incidence region for gastric cancer, accounting for 44.1% of all new cases and 49.9% of all deaths in the world (2). In China, the current 5-year survival rate for patients with gastric cancer is only 35.1% (3). The high heterogeneity of gastric cancer is reflected in the differences in histopathology, such as histological characteristics, cell differentiation degree, tissue occurrence, and cell growth pattern, as well as molecular features and immunologic expression characteristics (4). Within the same tumor, there are significant differences in tissue structure and cellular diversity (5), and the identification of HER2-overexpressing tumor subsets is a major advance in the treatment of gastric cancer (6, 7).

Human epidermal growth factor receptor 2 (HER2) is a tyrosine kinase receptor that promotes cancer cell differentiation, development, and survival (8). It belongs to the EGFR family and is involved in multiple metabolic and regulatory signaling pathways, including the PI3K/AKT/mTOR and RAS/RAF/MAP kinase pathways (9). Abnormal amplification and protein overexpression of the HER2 gene can lead to abnormal HER2 signaling in cancer cells, driving the oncogenic phenotype of HER2. In a preclinical model, it has been demonstrated that the amplification and activation of HER2 can drive cell transformation and tumor development, which is consistent with the etiology and clinical characteristics of certain cancers (10, 11).

HER2 gene amplification and protein overexpression were first reported in gastric cancer in 1986 (12, 13). Currently, HER2 overexpression is found in various cancers (14). It has been reported that HER2 overexpression accounts for approximately 12-20% of gastric cancer cases (15). In a recent meta-analysis of 41 studies (N=17494) on gastric cancer patients who were tested for HER2, the HER2 positivity rate was 19.07%. The subgroup analysis showed that the HER2 expression rate in Asia was 19.52%, higher than the rate in Europe (16.91%) (16). Another study revealed that the overexpression rate of HER2 in all Asian gastric cancer patients (n = 5301) was 9.7%, but was 18.1% when Chinese patients were excluded, indicating regional differences in the incidence rate (17). A meta-analysis of HER2 expression in gastric cancer has also found that intestinal-type gastric cancer, tumors located in the proximal part, and well-differentiated tumors are associated with a higher HER2 expression rate (16).

Trastuzumab is a monoclonal antibody and an antagonist of HER2. Clinical trials by Bang and Slamon have found that when trastuzumab is used in combination with chemotherapy to treat HER2-positive breast and gastric cancer patients, the objective response rate (ORR) is about 50%, and the median overall survival (mOS) exceeds 1-2 years (14, 18). In addition, it has been demonstrated that the addition of the anti-HER2 antibody pertuzumab, which targets different HER2 epitopes, increases ORR to 80% and mOS to 56.5 months in metastatic breast cancer patients treated with trastuzumab/chemotherapy (19, 20). Based on the significant results of clinical research, anti-HER2 therapy has been approved for first-line treatment of metastatic breast and gastric cancer patients.

US FDA-approved immunohistochemistry (IHC) and fluorescence *in situ* hybridization (FISH) tests are the standard methods for evaluating the status of HER2 expression. IHC is based on the antigen-antibody reaction; which is prone to non-specificity and is at most a semi-quantitative method. Due to its low cost, speed, and inexpensive equipment requirement, IHC is the preferred method for detecting HER2 expression status in routine pathological diagnosis work. In clinical practice, the HER2 IHC score is divided into three categories: negative (0+ or 1+), equivocal (2+), and positive (3+) (21, 22). Interpretation of IHC results by pathologists is subjective; as a result, there are certain false positive and false negative issues (14, 23). However, regarding whether IHC (or immunofluorescence, IF) can achieve accurate quantification, Toki et al. (24) conducted important experimental research. Epidermal growth factor receptor (EGFR) was measured by quantitative immunofluorescence (QIF) in 15 cell lines with a wide range of EGFR expression, using different primary antibody concentrations, including the optimal signal-to-noise concentration after quantitative titration. The experiment found that the best agreement between IF scores and LT-SRM absolute protein concentration was found when the EGFR D38B1 primary antibody was used at the optimal signal-to-noise concentration (0.017 µg/ml), showing a strong linear regression between the two assays (R^2^ = 0.88). It was also pointed out that the linearity of the agreement decreased when the working concentration moved away from the optimal concentration of the EGFR D38B1 primary antibody. Although all the results of this study come from cell line studies, and the clinical application value still needs further verification, it does answer the question of whether IHC or IF can achieve accurate quantification. Moreover, according to multiple reports, there are up to 20% false positive cases in patients judged HER2 positive by IHC, and false negative rates range from 1.1% to 11.5% in patients judged HER2 negative (25, 26). For HER2 IHC 2+ (equivocal results), FISH/ISH methods are recommended to confirm HER2 positivity. Currently, FISH is the gold standard for detecting HER2 gene amplification, and the guidelines define FISH/ISH positivity as a ratio of HER2 signals to centromere 17 signals (CEP17) ≥2.0 (27, 28). Several studies have suggested that the optimal threshold value is 4.0 (29, 30). Although IHC and FISH results are generally highly correlated, some researchers have found discrepancies and inconsistencies between the two methods (31), which may be caused by multiple factors, such as controlling gene signal changes, tumor heterogeneity, and technical errors (32).

The emergence of clinical mass spectrometry has aided in the advancement and growth of molecular diagnostic techniques. Mass spectrometry-based protein quantification is a novel method that has distinct advantages over conventional IHC diagnostic methods and overcomes the limitations of IHC and FISH methods. The advantages and disadvantages of IHC, FISH, and mass spectrometry-based selected reaction monitoring (MS-SRM) methods are compared in **Table S1**. MS-SRM enables absolute linear quantification of protein expression levels in tumor cells with HER2 expression levels greater than five orders of magnitude and simultaneous quantification of multiple protein biomarkers throughout the treatment process (33). MS-SRM technology is widely accepted for quantifying the protein expression levels in biological samples (34, 35).

The purpose of this study is to introduce a standardized MS-SRM method, aiming to establish a mass spectrometry targeted protein quantification platform for formalin-fixed, paraffin-embedded (FFPE) tumor tissue in China, and to systematically validate the workflow, including sample laser microdissection technology platform, detection linear range, limit of detection, assay accuracy, precision, and stability, etc. Subsequently, 118 FFPE sections of advanced gastric adenocarcinoma in China were retrospectively analyzed using this method to quantify the HER2 protein expression levels in the samples. Through a parallel comparison study of IHC and MS-SRM methods, we aim to accurately identify gastric patients with HER2 overexpression who will benefit from anti-HER2 treatment, thereby improving clinical treatment outcomes.

## Materials and methods

### Patient information and sample source

This study selected 118 paraffin specimens of gastric adenocarcinoma from 2015 to 2021, including 96 surgical specimens and 22 gastric biopsy specimens. The samples were obtained from the Central Hospital of Fushun City, Liaoning Province, Liaoning Cancer Hospital, and the First Affiliated Hospital of China Medical University. Patients who received neoadjuvant chemotherapy were excluded. The study cohort consisted of 6 HER2 negative samples (IHC 0), 14 cases of HER2 IHC 1+, 24 cases of HER2 IHC 2+/FISH-, 21 cases of HER2 IHC 2+/FISH+, and 53 cases of HER2 IHC 3+. The clinical and medical records of the patients were obtained from the hospital’s electronic medical record system, including gender, age, tumor size, degree of differentiation, WHO classification, Lauren classification, TNM staging, lymph node metastasis, and others. The entire sample met the requirements, and the basic characteristics and features of gastric adenocarcinoma patients are shown in **Table S2**. The study was approved by the Institutional Review Board of Fushun Central Hospital in Liaoning Province (ethical review number: 2021002).

### Evaluation of HER2 status in gastric adenocarcinoma - immunohistochemistry

**Figure 1:**
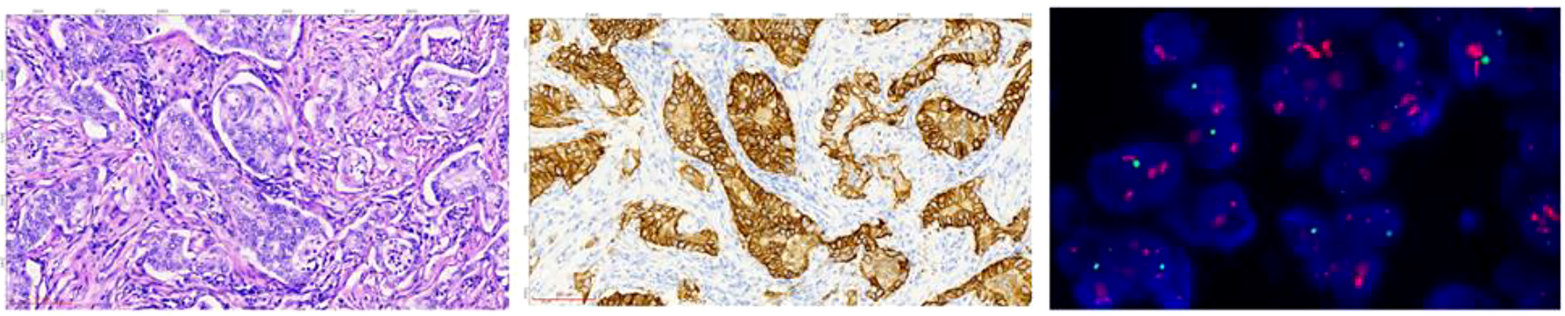
The HE, IHC, and FISH images of tissue sections from the same site of gastric adenocarcinoma of sample D185834. HE staining image (left): the cancer tissue is arranged in a glandular pattern, with large cancer cell nuclei that appear circular or oval (HE staining: 20X magnification). IHC staining image (middle): the cancer tissue staining shows positive cell membrane staining, presenting as a complete and strong circumferential staining, with a staining result of 3+ (EnVision staining: 20X magnification). FISH microscopy image (right): the test result was positive, and the HER2 gene showed clustered amplification. The red signal represents the HER2 gene, the green signal represents the centromere of chromosome 17, CEP17, and the blue signal represents the nucleus stained with 4’,6-diamidino-2-phenylindole (DAPI). The HER2 signal to CEP17 signal ratio was 13.5, indicating HER2 gene amplification. (FISH staining: 100X magnification).

IHC detection: The automatic immunohistochemistry staining instrument (model: UtraPATH) manufactured by China Zhongshan Golden Bridge Company was used for the detection, with both positive and negative controls set up for all cases. The HER2/NEU (4B5) antibody and DAB detection kit used were provided by China Zhongshan Golden Bridge Company. The HER2 IHC results were interpreted and scored by two pathologists in accordance with the Chinese Gastric Cancer HER2 Testing Guidelines (2016 edition) (36). The IHC diagnostic criteria for gastric adenocarcinoma surgical specimens were: 0, no reaction or <10% of tumor cell membrane staining; 1+, weak or faint membrane staining in ≥10% of tumor cells or only partial membrane staining; 2+, weak to moderate basolateral, lateral, or complete membrane staining in ≥10% of tumor cells; 3+, strong basolateral, lateral, or complete membrane staining in ≥10% of tumor cells. IHC scores of 0 and 1+ were negative, 2+ was indeterminate, and 3+ was positive. The diagnostic criteria for gastric biopsy specimens were: 0, no membrane staining in any tumor cells; 1+, weak or faint membrane staining in tumor cell clusters (regardless of the percentage of stained tumor cells in the entire tissue); 2+, weak to moderate basolateral, lateral, or complete membrane staining in tumor cell clusters (regardless of the percentage of stained tumor cells in the entire tissue, but with at least 5 clustered tumor cells stained); 3+, strong basolateral, lateral, or complete membrane staining in tumor cell clusters (regardless of the percentage of stained tumor cells in the entire tissue, but with at least 5 clustered tumor cells stained). See **Figure 1** (middle).

### Fluorescence *in situ* hybridization

FISH is used to detect the *HER2* gene amplification in specimens. The human *HER2* detection kit from Wuhan Kanglu Biological Technology Co. was used. The method involved tissue section dewaxing, dehydration, and denaturation, followed by denaturation and hybridization in an *in-situ* hybridization instrument (SH2000, Hangzhou Ruicheng Instrument Co., Ltd.). After counterstaining, the slides were examined under a microscope (Axio Scope.AI, Zeiss) and interpreted according to the standards. For gastric cancer specimens, the HER2 FISH interpretation standard was negative if HER2/CEP17 <1.8, and positive if HER2/CEP17 ≥2.2 or when the signal clusters. If 1.8≤*HER2*/*CEP17* <2.2, 20 cells were recounted and positive if the ratio was ≥2.0, and negative if the ratio was <2.0. Refer to **Figure 1** (right) for details.

### Laser microscope for tumor cell cutting and sample preparation

The tissue specimens were fixed in 10% neutral formalin for 6-24 hours. Eight consecutive slices, including one 4 µm slice for HE staining, shown in **Figure 1** (left), one 3 µm slice for IHC testing, one 3 µm slice for FISH testing, and five 10 µm slices for MS-SRM testing were prepared from the tissue block. IHC and FISH were tested using routine methods (omitted).

### Tumor cell annotation and collection

Tumor cell cutting was performed by taking 4 µm and 10 µm thick FFPE slices from each sample followed by staining them with hematoxylin and eosin. The digital pathology scanning system (3DHistech, MIDI) scanned the H&E and 10 µm slices and images, and a pathologist marked specific tumor cell regions (≥8 mm2) on the images. The slices were then placed on the laser microdissection instrument (Nikon, eclipse Ni-U) stage, and the marked images were imported into the instrument system for laser microdissection. The tumor cells on the slides were separated and collected into an Eppendorf tube using laser energy.

### Lysis of tumor cells

The collected tissue pellets were dried and then processed using a Liquid Tissue® protocol and the resultant peptides are quantified using a micro-BCA method (Thermo Fisher Scientific, 23231).

### Sample preparation for mass spectrometry

The tumor lysate peptides were mixed with isotopically labeled HER2 standard peptide and injected into a liquid chromatography-mass spectrometry (LC-MS) platform for HER2 quantification. Each injection resulted in 5000 amol of heavy internal standard peptide and 1µg total protein on-column.

### HER2-SRM analysis method

HER2-SRM analysis was performed on a liquid chromatograph (LC) (Waters ACQUITY UPLC M-Class System) connected to a triple quadrupole mass spectrometer (Thermo TSQ-Altis).

An LC gradient was use used to elute peptides. The flow phase A was water with 0.1% formic acid (Thermo Scientific, LS118), and the flow phase B was acetonitrile with 0.1% formic acid (Thermo Scientific, LS120). The chromatographic column set included a trap column (nanoEase MZ Symmetry C18 Trap Column, 100A, 5 µm 180 µm x 20mm) and an analytical column (nanoEase MZ HSS T3 Column, 100Å, 1.8 µm, 100 µm x 100 mm).

Peptides were eluted into the mass spectrometer using the following gradient: loaded onto trap column for 5 min with buffer A at a flow rate of 5 µl/min and eluted with buffer B using a step gradient at 800 nl/min. Buffer B was increased from 1–25% (8 min), 25–50% (7 min), and 50–95% (3 min). Finally, the column was cleaned with buffer B for 6 min and equilibrated with buffer A for 4 min.

Mass spectrometry method: Thermo TSQ-Altis mass spectrometer was operated in positive NSI mode and were used for the SRM assays: Q1(FWHM):0.7, Q3(FWHM): 0.7, dwell time: 100 ms, electrospray voltage: 2.3 kV, collision gas: 2 mTorr. The precursor ions for the light and heavy peptides are m/z 483.748 and 488.752. The fragment ions for the light and heavy peptides and their corresponding optimized collision energy are m/z 409.218 (17V)/538.261 (17V)/625.294 (17V) and 419.227 (17V)/548.270 (17V)/635.302 (17V), respectively.

### Mass spectrometry data acquisition and processing

The area under the curve (AUC) for the endogenous peptide and for isotopically labeled standard peptide was used to calculate peptide quantity by Pinnacle Production software (version number: V 1.0.83.0) and the data collated by Microsoft Excel. According to the AUC of endogenous HER2 peptide and heavy peptide, the concentration of endogenous peptide for HER2 for each sample was calculated using the following formula:


$$\begin{array}{c} \text{Quantity of HER}2\left(\text{amol}/\text{µg}\right)\\ =\left(\text{AUC of endogenous peptide}/\text{AUC of isotopically labeled standard peptide}\right)\\ \times \left(\text{amol of isotopically labeled peptide}*/\text{µg total protein}\right) \end{array}$$

where labeled peptide* equals quantity of spiked isotopically labeled standard (amol) injected and total protein equals quantity of total protein injected.

### Statistical strategy and statistical processing method

Data Collection: In addition to collecting relevant clinical information, HER2 protein expression by MS-SRM and IHC, and HER2 gene expression levels was included in the statistical analysis data. Statistical analysis of baseline data of study subjects is performed using R 4.2.2 version. The statistical analyses in this study included several commonly used tests to examine the differences between groups among variables. The t-test was used to compare means between two groups, assuming that the data were normally distributed and had equal variances. The Wilcoxon rank sum test was used as a non-parametric alternative to the t-test when the normality assumption was not met. The chi-squared test was used to examine the association between two categorical variables.

## Results

### Development of HER2-SRM targeted protein mass spectrometry quantification method

#### Selection and quantification of HER2 protein characteristic peptides

**Figure 2:**
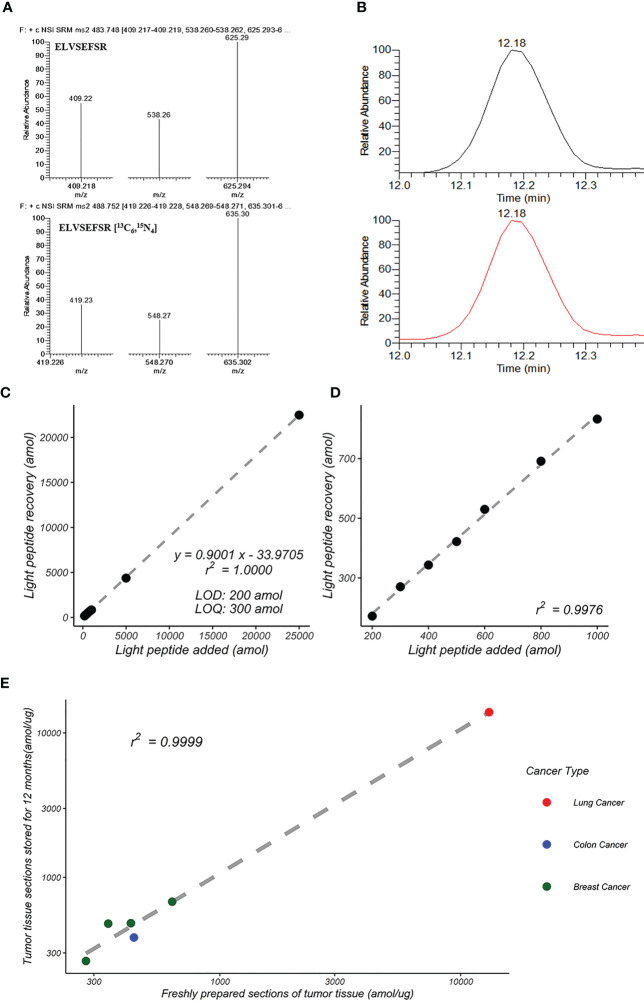
Development of HER2-SRM quantification method. **(A)** MS/MS spectra of ELVSEFSR and ELVSEFSR [13C6,15N4]. **(B)** Total ion chromatograms (TIC) of ELVSEFSR and ELVSEFSR [13C6,15N4]. **(C)** Standard curves of peptide input and recovery for HER2-SRM method. It shows the linear relationship over a concentration range of 200 amol/µg to 25,000 amol/µg, and **(D)** shows the linear relationship over a concentration range of 200 amol/µg to 1,000 amol/µg. **(E)** Reproducibility of HER2-SRM method for the same FFPE sample after 12 months. Green represents breast cancer samples (n=4), red represents lung cancer samples (n=3), and blue represents colon cancer samples (n=2).

The literature indicates that the single unique peptide (ELVSEFSR) gave the most reproducible detection with the highest intensity in both trypsin-digested recombinant HER2 and FFPE tumor tissues (37). So, we selected “ELVSEFSR” (light peptide) as the HER2 peptide for the development and validation of the HER2-SRM method. The isotope-labeled peptide ELVSEFSR [^13^ C _6,_^15^ N _4_] (heavy peptide) was used as the internal standard. The transition ions of each peptide are shown in **Figure 2A**; the elution curve, retention time and total ion chromatograms are shown in **Figure 2B**.

### Linearity and accuracy of the HER2-SRM mass spectrometry analysis method

**Figure 3:**
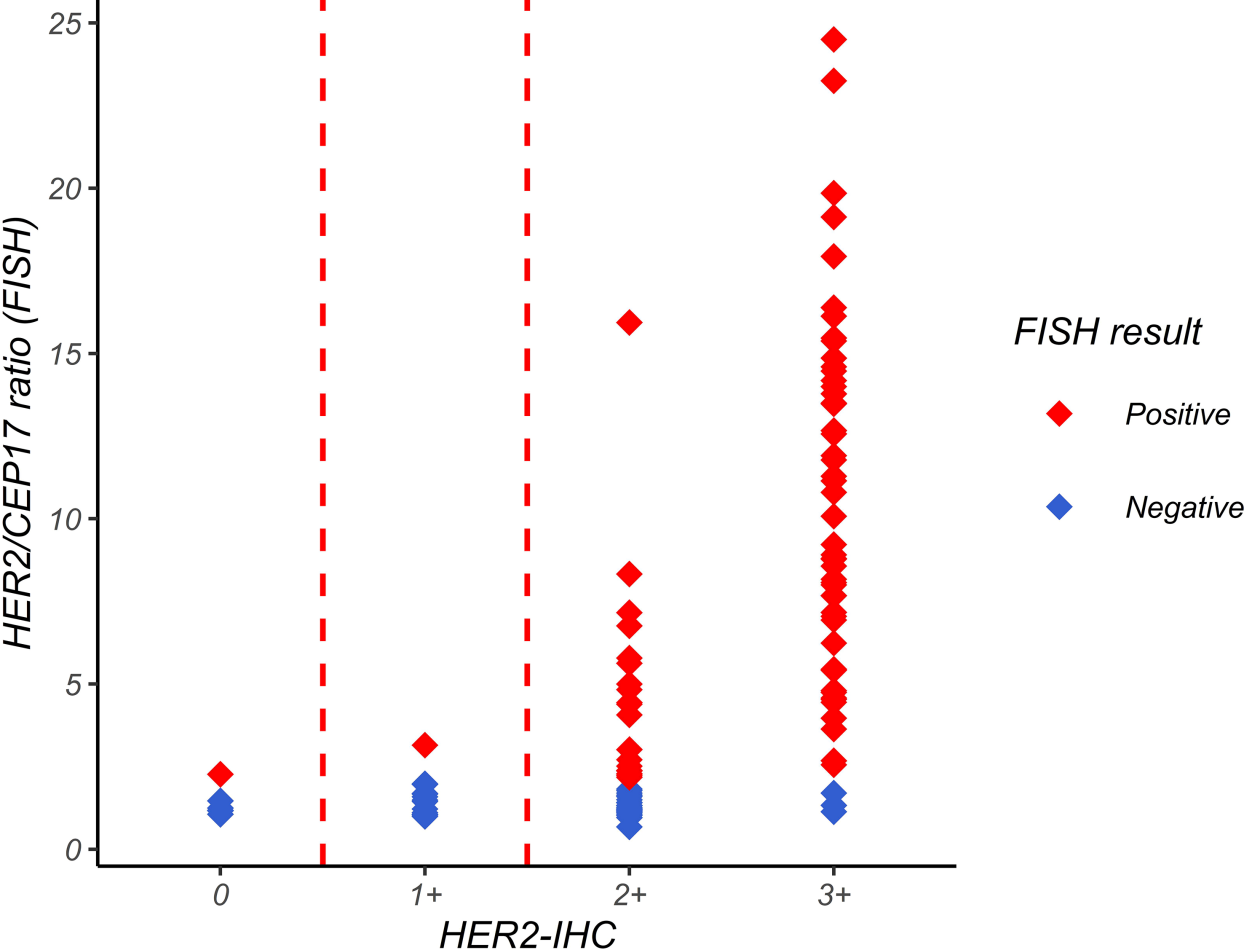
Relationship between *HER2/CEP7* ratio by FISH and IHC interpretation. The horizontal axis represents the IHC interpretation result, and the vertical axis represents the HER2/CEP17 ratio in FISH detection. Each point in the figure represents a sample point. The figure shows that the average level of HER2/CEP17 ratio is lower when the IHC interpretation result is 0 or 1+, and higher when the IHC interpretation result is 3+.

Standard curve samples were prepared using a complex proteome standard matrix (P. furiosus coccus enzymatic solution, CPS). The range of the curve is from 50 amol/µg to 25000 amol/µg. Five replicates were analyzed for each concentration point. Test results: (1) The limit of detection (LOD) is 200 amol/µg, and the limit of quantification (LOQ) is 300 amol/µg, (2) Linear range from 300 amol/µg to 25000 amol/µg, linear regression value r^2 ^= 1.000, see **Figures 2C, D**, (3) Coefficient of variation (CV%) of the five replicate samples ranged from 0.7% to 9.6%, and (4) The accuracy range for each concentration is between 83.2% and 90.1%. The linearity, accuracy and small CV% demonstrated that the method is accurate and reproducible.

#### Precision verification of the HER2-SRM method

##### Intra-day precision of the HER2-SRM method

To evaluate the intra-day precision of the HER2-SRM method, four concentrations of quality control samples (500 amol/μg, 1000 amol/μg, 5000 amol/μg, and 10000 amol/μg) were prepared using the CPS, and injected five times for each concentration. Twenty quality control samples were continuously tested within 1 day, and the CV% of the quality control samples was less than 10%, ranging from 2.5% to 9.7%.

##### Inter-day precision of the HER2-SRM method

The inter-day precision of the HER2-SRM method was evaluated over 20 days. The evaluation method involved analyzing four concentrations of quality control samples (500 amol/μg, 1000 amol/μg, 5000 amol/μg, and 10000 amol/μg) daily for 20 days using the HER2-SRM method and calculating the CV% of the four concentration quality control samples over the 20-day period. The results showed that the CV% of the quality control samples over the 20-day period were less than 10%, ranging from 3.4% to 9.7%.

### Reproducibility verification of the HER2-SRM method

We used nine tumor FFPE samples (2 colon adenocarcinomas, 4 breast cancers, and 3 lung cancers) to verify the reproducibility of the HER2-SRM method. Two tumor FFPE sections from the same sample were compared 12 months apart. The results showed good consistency between the detection results of freshly prepared tumor tissue slices and those stored for 12 months, with a linear correlation coefficient of 0.9999, as shown in **Table S3** and **Figure 2E**.

### Comparison of HER2-IHC and *HER2*-FISH testing results

A correlation analysis of the IHC and FISH results of 118 FFPE slices was conducted. In the IHC scoring, 0 and 1+ were considered negative, 2+ as equivocal, and 3+ as positive. As shown in **Figure 3**, the average level of the HER2/CEP17 ratio was lower when the IHC reading result was 0 or 1+. The average ratio of HER2/CEP17 was in the middle when the IHC reading result was 2+. The average level of the HER2/CEP17 ratio was higher when the IHC reading result was 3+. This suggests that IHC and FISH are consistent to some degree.

### Comparison of HER2-IHC and HER2-SRM test results

**Figure 4:**
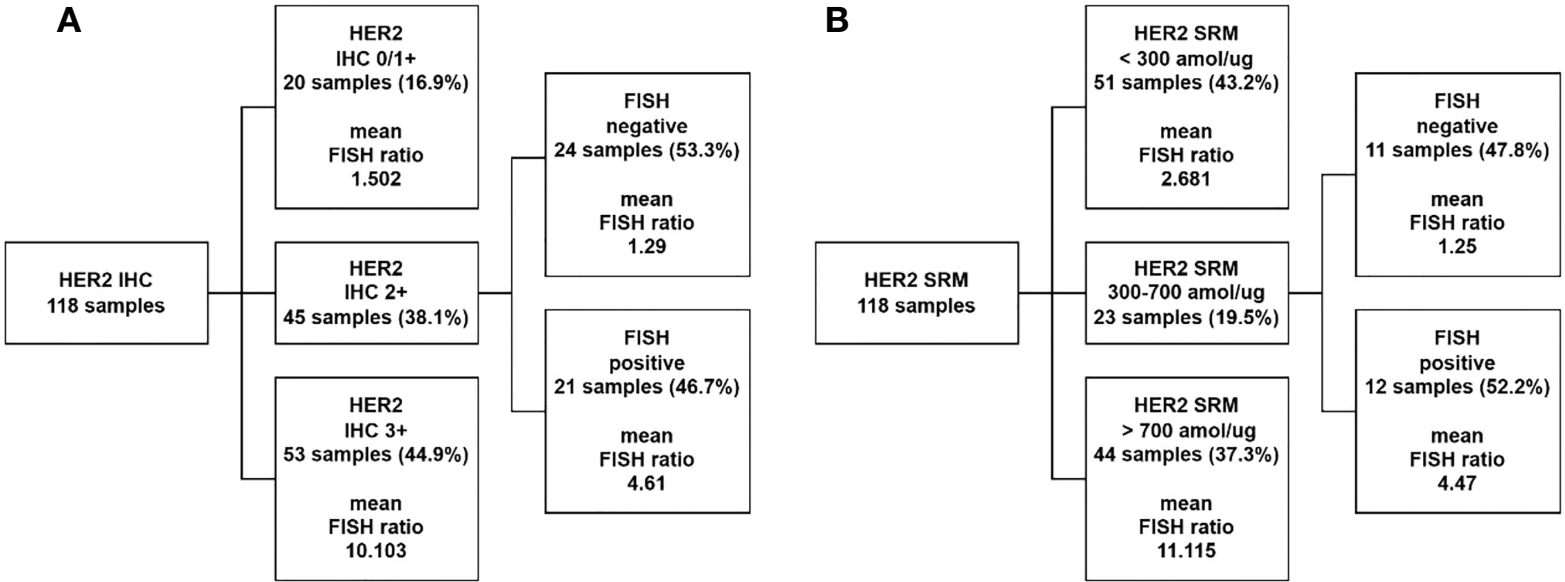
Comparison of classification performance between HER2-IHC and HER2-SRM methods. **(A)** 118 samples were tested using the HER2-IHC method, where 20 samples (represented by the second layer box one) with HER2-IHC scores of 0/1+ were classified as negative, accounting for 16.9% of the total study samples. The average FISH ratio of these samples was 1.502. 45 samples (represented by the second layer box two) with HER2-IHC scores of 2+ required FISH testing for interpretation, accounting for 38.1% of the total study samples. Among these 45 samples, 53.3% were classified as negative (represented by the third layer box one) and 46.7% were classified as positive (represented by the third layer box two) by FISH testing. 53 samples (represented by the second layer box three) with HER2-IHC scores of 3+ were directly classified as HER2 positive by IHC testing, accounting for 44.9% of the total study samples. The average FISH ratio of these samples was 10.103. **(B)** 118 samples were tested using the HER2-SRM method, where 51 samples (represented by the second layer box one) with HER2-SRM expression levels below the lower limit of detection (300amol/µg) were directly classified as HER2 negative, accounting for 43.2% of the total study samples. The average HER2-FISH ratio of these samples was 2.681. 23 samples (represented by the second layer box two) with HER2-SRM expression levels between the upper and lower limits of detection (300-700amol/µg) accounted for 19.5% of the total study samples. Among these 23 samples, 47.8% were classified as negative by HER2-FISH testing (represented by the third layer box one), and 52.2% were classified as positive by HER2-FISH testing (represented by the third layer box two). 44 samples (represented by the second layer box three) with HER2-SRM expression levels above the upper limit of detection (700amol/µg) were directly classified as HER2 positive, accounting for 37.3% of the total study samples. The average HER2-FISH ratio of these samples was 11.115.

HER2-IHC was used to classify the 118 study samples, and the results are shown in **Figure 4A**. Among them, 20 samples were classified as IHC 0/1+ and were directly classified as HER2-negative by HER2-IHC. 45 samples were classified as IHC 2+ and could not be categorized as HER2-positive or HER2-negative by HER2-IHC. 53 samples were classified as IHC 3+ and were directly classified as HER2-positive by HER2-IHC.

HER2-SRM was used to classify the 118 study samples, and the results are shown in **Figure 4B**. Among them, 51 samples had HER2-SRM expression levels below the limit of quantification of 300 amol/µg and as shown later were directly classified as HER2-negative by HER2-SRM, and their average HER2-FISH ratio was also lower, showing good consistency between HER2-SRM and *HER2-FISH*. In the 118 samples, only 23 samples had HER2-SRM expression levels between the upper and lower detection limits of HER2-SRM and could not be judged as positive or negative by HER2-SRM. In HER2-IHC, there were 45 such samples. Among the 118 samples, 44 samples had HER2-SRM expression levels above 700 amol/µg and as shown later were directly classified as HER2-positive by HER2-SRM, and their average HER2-FISH ratio was also higher, indicating good consistency between HER2-SRM and HER2-FISH.

Comparing the two methods, it was found that there were 45 samples (38.1% of the total) that could not be directly judged by the HER2-IHC method, while only 23 samples (19.5% of the total) with HER2-SRM quantitative values between 300 and 700 amol/µg were not directly judged by the HER2-SRM method, as defined later, which was almost twice as low as that of HER2-IHC. This reduces the number of samples that need to be confirmed by HER2-FISH testing. If samples that could not be directly judged by the HER2-SRM method and those that could not be directly judged by the IHC method were excluded, the sensitivity of HER2-IHC was 68.5%, and the specificity was 40.0%. The sensitivity of HER2-SRM was 60.3%, and the specificity was 75.6%. The two methods had similar sensitivity, but the specificity of HER2-SRM was significantly higher than that of HER2-IHC.

### HER-SRM and FISH results in IHC 2+ samples

**Figure 5:**
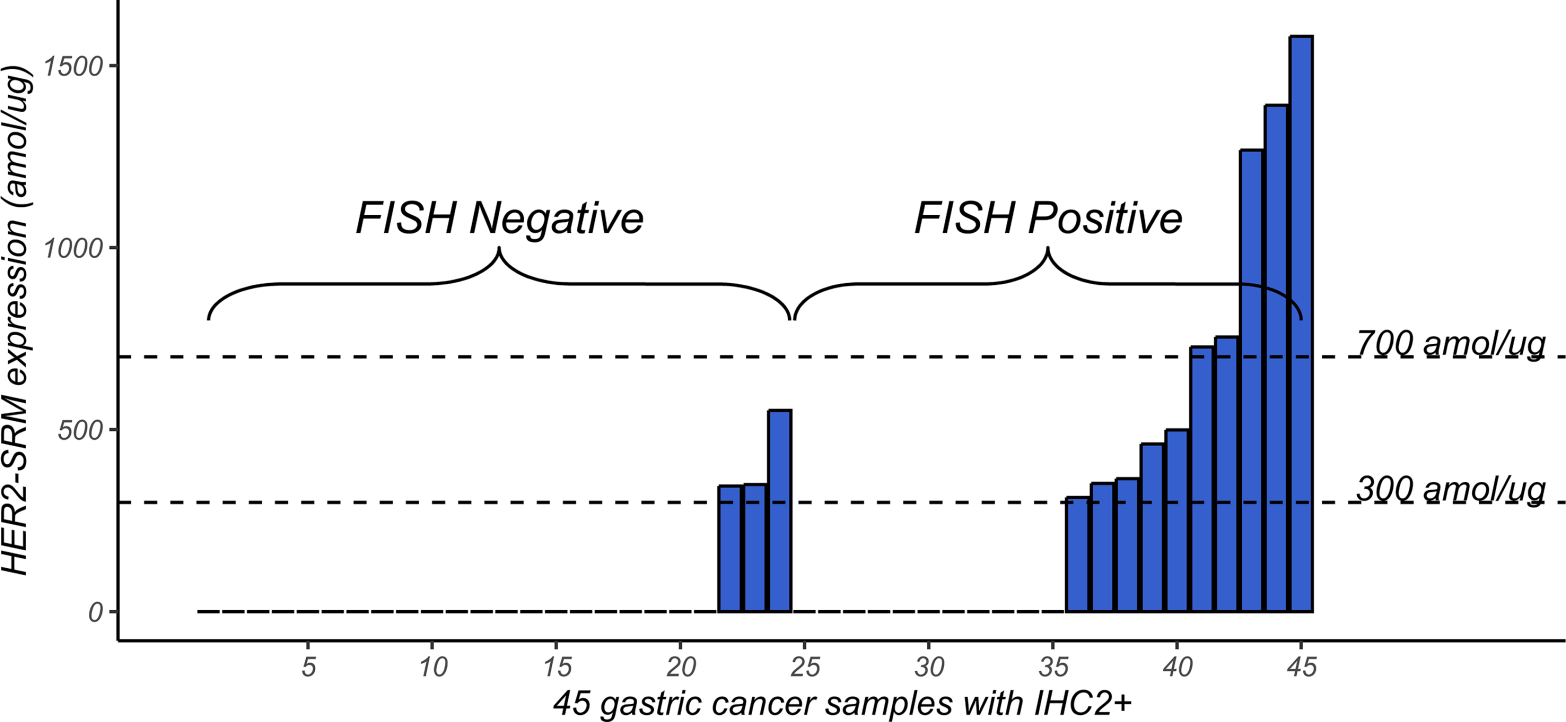
Distribution of HER2-SRM expression levels in 45 samples that tested IHC2+. The left-hand side of the horizontal axis shows the 1-24 FISH-negative samples, arranged from left to right according to the HER2-SRM expression level. The right-hand side of the horizontal axis shows the 25-45 FISH-positive samples, also arranged from left to right according to the HER2-SRM expression level. The height of the column represents the magnitude of the HER2-SRM expression level. From the figure, it can be seen that among these 45 IHC2+ samples, samples with high HER2-SRM expression levels are more likely to be found in the FISH-positive range.

As shown in **Figure 5**, among the 45 IHC 2+ samples, samples with high expression of HER2-SRM were more likely to appear in the FISH-positive range, but many samples with low expression of HER2-SRM also appeared in this range. Samples with low expression of HER2-SRM were more likely to appear in the FISH-negative range. This indicates that there is some consistency between FISH and HER2-SRM in classifying IHC 2+ samples, but there are also differences.

### Comparison of HER2-FISH and HER2-SRM results

**Figure 6:**
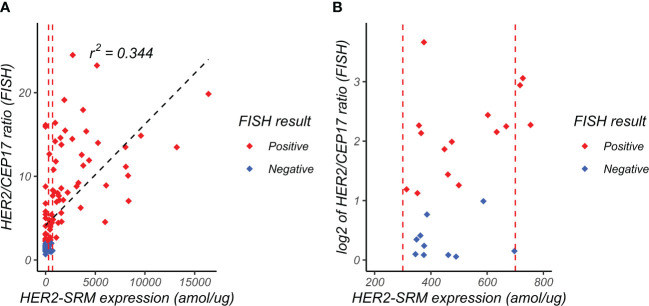
Relationship between FISH HER2/CEP17 ratio and HER2-SRM expression level. **(A)** the horizontal axis represents the expression level of HER2-SRM (amol/µg), and the vertical axis represents the FISH-determined HER2/CEP17 ratio. Each point in the figure represents a sample. From the figure, it can be seen that there is a positive correlation (r^2^ = 0.344) between HER2-SRM expression level and HER2/CEP17 ratio, indicating good consistency between the HER2-SRM and FISH methods. The red dashed line in the figure represents the selected lower threshold (300 amol/µg) and upper threshold (700 amol/µg). The black dashed line represents the calculated linear regression curve, and the linear regression equation is also shown. **(B)** is a locally magnified scatter plot of the relationship between HER2-SRM expression level and HER2/CEP17 ratio (FISH). It can be seen that within the range of 300-700amol/µg, both FISH-negative and FISH-positive sample points are relatively evenly distributed along the x-axis, and the projections of the positive and negative samples on the x-axis overlap completely and cannot be distinguished. On the y-axis, it can be seen that some samples with high HER2-FISH expression levels have low HER2-SRM expression levels, tending towards low expression.

As shown in **Figure 6**, the horizontal axis represents the expression level of HER2-SRM (amol/µg), and the vertical axis represents the ratio of HER2/CEP17 detected by FISH. Each point in the figure represents a sample. **Figure 6A** includes 118 samples included in the study. It can be seen that there is a moderate positive correlation between HER2-SRM expression and the ratio of HER2/CEP17 (Pearson correlation coefficient, r=0.586). The two vertical dashed lines represent the upper and lower limits of HER2-SRM classification. In order to better display the distribution of samples near the upper and lower limits, the horizontal axis of **Figure 6A** was expanded around 500 amol/µg, which is shown in **Figure 6B**.

### Relationship between HER2-SRM expression, IHC interpretation, and HER2/CEP17 ratio by FISH

**Figure 7:**
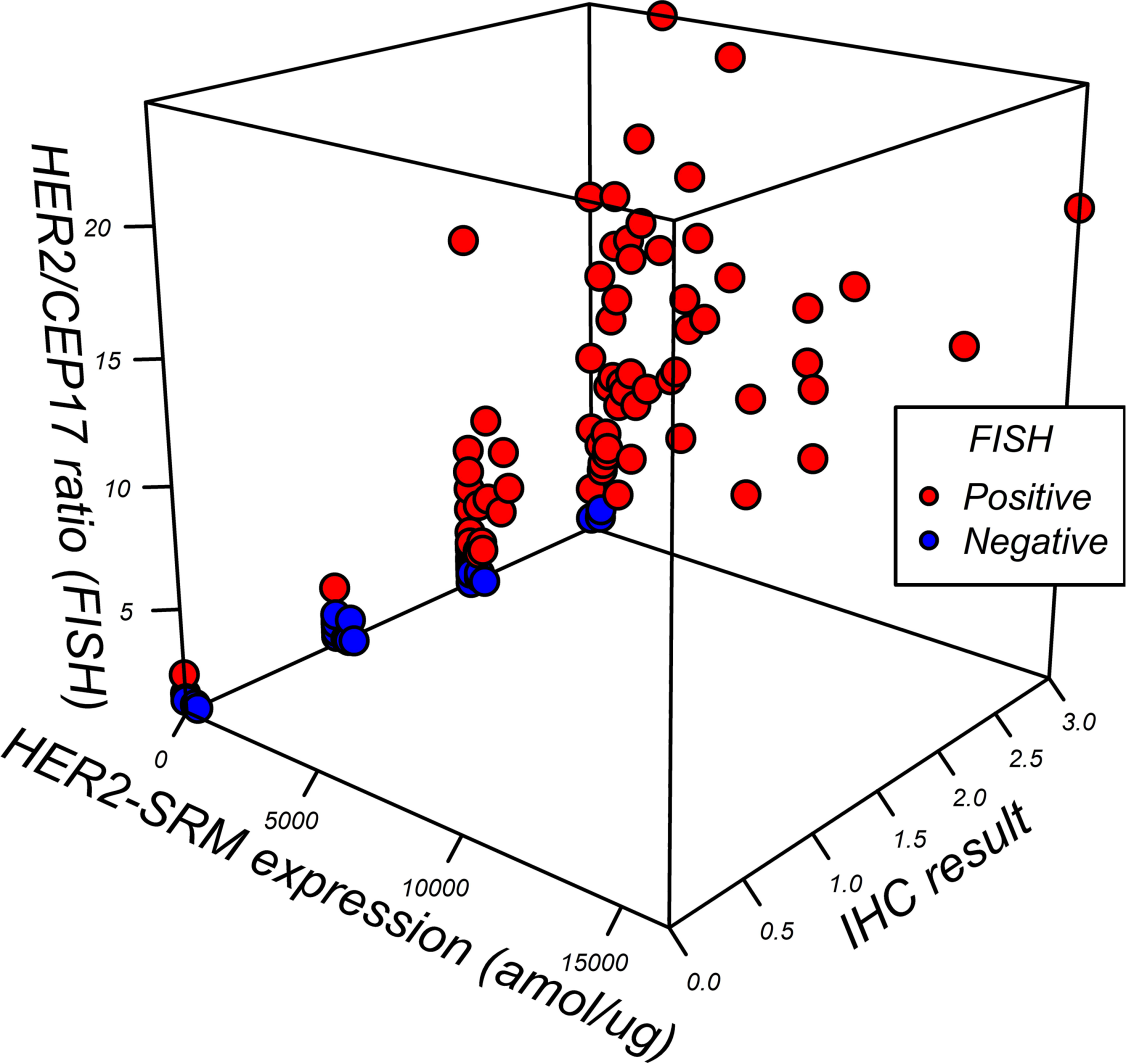
Relationship between HER2-SRM expression level, IHC interpretation, and FISH HER2/CEP17 ratio. Each point represents a sample, with a total of 118 samples. The three coordinate axes represent the FISH HER2/CEP7 ratio, HER2-SRM expression level, and IHC result, respectively. Red points represent FISH-positive samples, and blue points represent FISH-negative samples. From the figure, it is easy to see that the higher the HER2-SRM expression level, the higher the FISH HER2/CEP17 ratio, and the higher the IHC-positive rate (IHC3+).

**Figure 7** shows the relationship among HER2-SRM expression, IHC interpretation, and HER2/CEP17 ratio by FISH, and the higher the HER2-SRM level, the higher the HER2/CEP17 ratio detected by FISH and the higher the rate of IHC positivity (IHC 3+). However, there are individual samples with low expression of HER2-SRM but high HER2/CEP17 ratio. Overall, the directions of judgment for HER2-IHC, FISH, and HER2-SRM are consistent.

### Receiver operating characteristic curve (with FISH results as the actual category)

**Figure 8:**
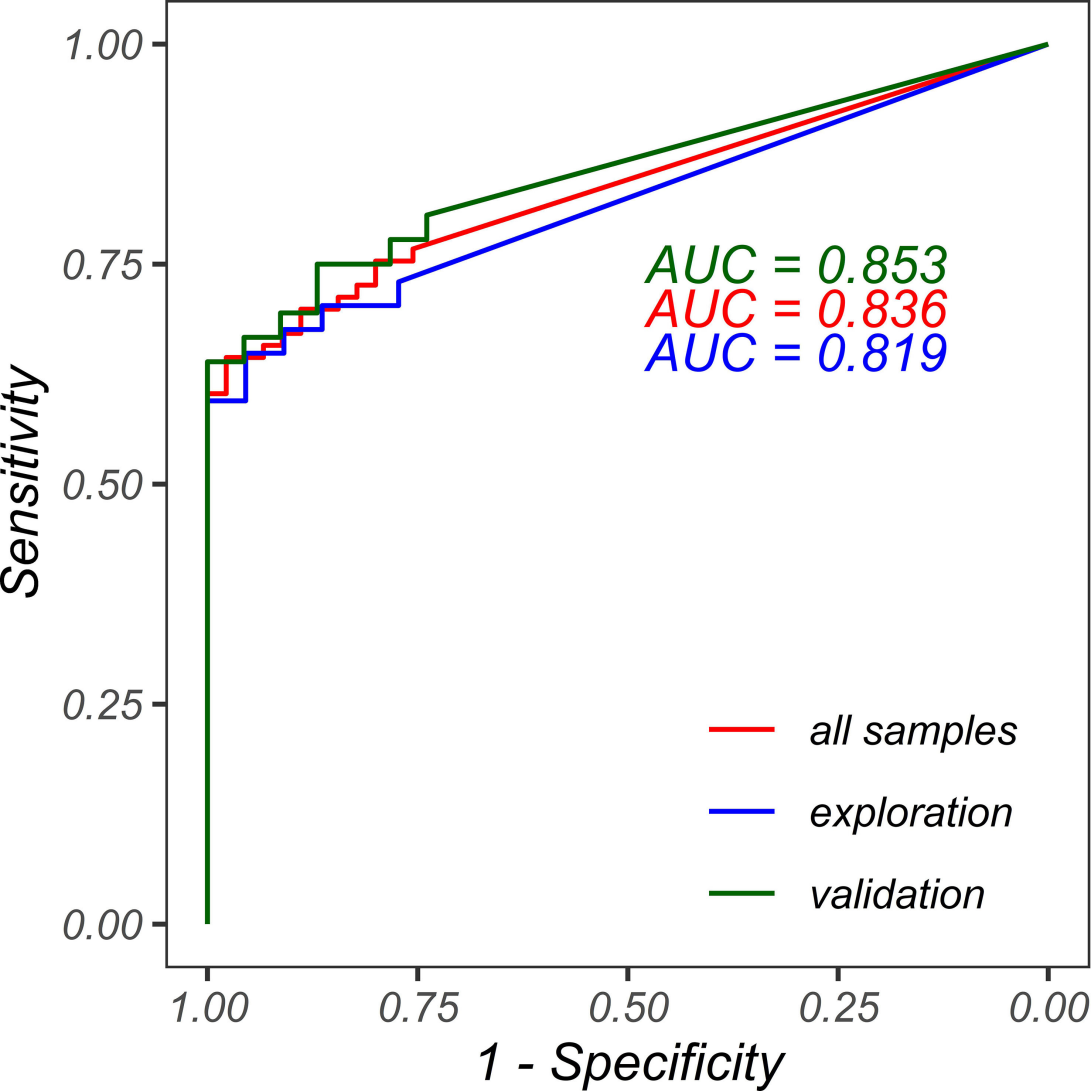
ROC curves for FISH classification using HER2-SRM protein expression. The red line represents the ROC curve for all samples with an AUC of 0.836. The blue line represents the ROC curve for the exploration set using HER2-SRM protein expression for FISH classification with an AUC of 0.819. The green line represents the ROC curve for the validation set using HER2-SRM protein expression for FISH classification with an AUC of 0.853.

With the assistance of ROC curve (the red curve shown in **Figure 8**) and with FISH results as the actual category, optimized Her2-SRM upper and lower limits were determined, HER2-SRM protein expression level has a high specificity of 100% and a sensitivity of 60.3% at 700 amol/µg, and has a specificity of 75.6% and a sensitivity of 76.7% at 300 amol/µg. Several different upper and lower limits were compared, and the threshold that could balance specificity and sensitivity was selected. Therefore, when using mass spectrometry quantification to detect HER2 protein expression in gastric cancer, 700 amol/µg can be used as the upper threshold and 300 amol/µg as the lower threshold.

Statistical analysis was performed based on the discrimination results of HER2-FISH as the disease and control groups, as shown in **Table S4**. Wilcoxon Rank Sum Test was performed on HER2-IHC, FISH, HER2/CEP17 ratio, HER2-SRM expression level, and TNM staging. χ2 test was performed on FISH interpretation results, gender, Lauren classification, lymph node metastasis, degree of differentiation, and tumor location. T-test was performed on age. The significance levels (p-values) of age, gender, Lauren classification, lymph node metastasis, TNM staging, degree of differentiation, and tumor location were all greater than 0.05, indicating no significant differences.

### Validating the accuracy of the upper and lower limits of HER2-negative and positive determination in the HER2-SRM method

To validate the accuracy of the upper and lower limits (300-700 amol/µg) for HER2-negative and positive determination in the HER2-SRM method, we divided the 118 study samples into two parts according to the sampling time. Fifty-nine samples collected before December 31, 2019, were used as the exploration set, and fifty-nine samples collected after December 31, 2019, were used as the validation set. If the upper and lower limits determined in the exploration set were the same or similar to the results obtained in the analysis of the 118 samples, and the performance was verified in the validation set, it could be considered that the upper and lower limits (300-700 amol/µg) for HER2 determination in the current HER2-SRM method have good accuracy and stability.

Data analysis and drawing in the exploration set: The ROC curve (blue curve in **Figure 8**) of HER2-SRM expression level for determining HER2-FISH positive and negative was analyzed. The results showed that the HER2-SRM expression level had high specificity (100%) and a sensitivity of 59.5% at the detection upper limit of 700 amol/µg, which was consistent with the detection upper limit results obtained from the 118 samples. At the detection lower limit of 300 amol/µg, it had a specificity of 77.3% and a sensitivity of 73.0%, which was consistent with the detection lower limit obtained from the 118 samples. At the same time, in the exploration set, the number of samples that could not be directly interpreted using the HER2-SRM method accounted for 16.9% of the total samples, which was similar to the 19.5% of samples that could not be directly interpreted from the 118 samples. In the validation set, in samples where HER2-negative and positive could be confirmed using the HER2-SRM method, the sensitivity (true positive rate, TPR) was 59.5% and the specificity (true negative rate, TNR) was 77.3%, which was similar to the sensitivity and specificity obtained from the 118 samples.

Data analysis and drawing in the validation set: The ROC curve (green curve in **Figure 8**) of HER2-SRM expression level for determining HER2-FISH positive and negative was analyzed. If a detection upper limit of 700 amol/µg was used, it had a specificity of 100% and a sensitivity of 61%, which was consistent with the specificity and sensitivity obtained from the 118 samples. If a detection lower limit of 300 amol/µg was used, it had a specificity of 73.9% and a sensitivity of 80.6%, which was also consistent with the specificity and sensitivity obtained from the 118 samples. Moreover, the number of samples that could not be directly interpreted using the HER2-SRM method accounted for 22.0% of the total samples, which was similar to the 22.0% of samples that could not be directly interpreted from the 118 samples. At the same time, in samples where HER2-negative and positive could be confirmed using the HER2-SRM method in the validation set, the sensitivity (TPR) was 61.1% and the specificity (TNR) was 73.9%, which was similar to the sensitivity and specificity obtained from the 118 samples. Thus, it can be seen that the upper and lower limits (300-700 amol/µg) for HER2-negative and positive determination in the HER2-SRM method have good accuracy and stability.

To verify the reasonable grouping of the exploration set and validation set, statistical analysis was performed on the two parts using Wilcoxon Rank Sum Test for HER2-IHC, HER2-FISH, HER2/CEP17 ratio, HER2-SRM expression level, and TNM staging, and using χ2 test for FISH results, gender, Lauren classification, lymph node metastasis, differentiation degree, and tumor location. The significant levels (p-values) of each variable were all greater than 0.05, indicating no significant differences and reasonable grouping, as shown in **Table S5**.

### Comparison of HER2-IHC, FISH, and SRM methods in 22 gastric adenocarcinoma biopsy samples

In some Chinese gastric cancer patients, the disease is diagnosed at an advanced stage, making surgical resection impossible, and only tissue biopsy can be used to evaluate the expression status of HER2. It is clinically significant to accurately identify and select individuals who can benefit from anti-HER2 therapy. The clinical value of predictive biomarker detection depends on the stability, sensitivity, and specificity of the detection methodology. We have actively explored this area. The clinical basic conditions and tumor characteristics of 22 gastric adenocarcinoma biopsy samples are shown in **Table S6**.

In the 22 gastric adenocarcinoma biopsy samples, there was one case with FISH negative and SRM detection value less than 300 amol/µg sample, and 11 cases with FISH positive and SRM detection value greater than 700 amol/µg sample. The consistency rate between SRM detection results and FISH results was 54.55%, as shown in **Table S7**. Our preliminary research results are similar to those of some researchers (32), but contrary to those of others (38).

### Preliminary clinical study of accurate subtyping (grouping) of gastric adenocarcinoma mixed type (Lauren classification) patients based on HER2-SRM method

Taking HER2 expression (IHC 1+, 2+, 3+) of gastric adenocarcinoma mixed type (Lauren classification) patients as the research object, we conducted a preliminary clinical study of accurate subtyping (grouping) based on Lauren classification and referring to the intestinal and non-intestinal phenotypic characteristics in the mixed type according to the WHO classification.

First, 28 mixed-type (Lauren classification) samples were pathologically reexamined from 96 surgical gastric adenocarcinoma samples, and one case was found to be a microsample, which was excluded from the study, leaving 27 samples in total. The 27 samples were divided into two groups: 11 samples with relatively typical tubular adenocarcinoma intestinal structure features and 16 samples without typical tubular adenocarcinoma intestinal structure features, as shown in **Table S8**.

### Establishment and statistical analysis of the mixed-type intestinal and non-intestinal phenotypic feature subtyping model

**Figure 9:**
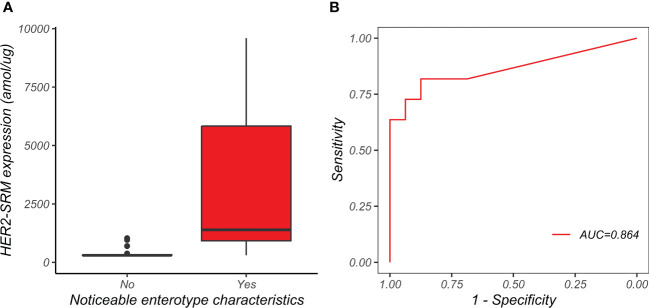
The relationship between the presence of intestinal phenotype and HER2-SRM expression in mixed type samples. **(A)** the vertical axis represents the HER2-SRM protein expression detected by mass spectrometry in mixed type gastric cancer patient samples, and the horizontal axis represents whether the patient samples have an intestinal phenotype. **(B)** the ROC curve for the use of HER2-SRM protein expression to determine whether mixed type gastric cancer samples have an intestinal phenotype is shown, with an AUC of 0.864.

In **Figure 9A**, the y-axis represents the HER2-SRM expression level detected by mass spectrometry in gastric adenocarcinoma mixed-type patient samples, and the x-axis represents whether the samples have intestinal phenotypic features. It can be seen that the HER2-SRM expression level in mixed-type patient samples with intestinal phenotypic features is significantly higher than that in mixed-type patient samples without typical intestinal phenotypic features, and the overlap between the two on the y-axis is small, providing a basis for using the HER2-SRM expression level as a linear mathematical model to distinguish between the two groups of patients.

Using the expression level of HER2-SRM as a linear model to predict and determine whether mixed-type gastric adenocarcinoma (Lauren classification) has intestinal phenotype features through ROC curve analysis (as shown in **Figure 9B**), the results indicate that HER2-SRM expression level has a high classification performance in the pathological diagnosis of mixed-type gastric cancer with intestinal phenotype features, with an AUC of 0.864. Based on the assumption that the pathological judgment of mixed-type gastric cancer with intestinal phenotype features is good, after weighing and selecting different judgment criteria for sensitivity and specificity, we believe that the specificity and sensitivity of HER2-SRM protein expression detection level are good when judged at 700 amol/µg, with a specificity of 87.5% and a sensitivity of 81.8%. As shown in **Table S9**, apart from the total rows and columns, the sum of the numbers on the diagonal from top left to bottom right represents the cases where HER2-SRM and pathological diagnosis are consistent, while the sum of the numbers on the diagonal from top right to bottom left represents the cases where HER2-SRM and pathological diagnosis are inconsistent. Among the 27 study samples, there were only 4 cases of disagreement between HER2-SRM and pathological diagnosis, and the remaining 23 cases were accurately predicted, showing the good accuracy of HER2-SRM method in determining the pathological diagnosis of whether mixed-type gastric adenocarcinoma (Lauren classification) has intestinal phenotype features. Therefore, we define 700 amol/µg as a judgment threshold for determining the intestinal and non-intestinal types in mixed-type (Lauren classification) gastric cancer patients based on mass spectrometry HER2-SRM quantitative detection of gastric cancer HER2 protein expression.

## Discussion

Numerous clinical studies have shown that HER2 plays an important role in the development of gastric cancer (6, 38–40). HER2 positivity in gastric cancer is associated with a worse prognosis, increased disease invasiveness, and decreased survival (41, 42). However, some studies have reported that HER2 status does not have prognostic value (42). We believe that these inconsistencies are primarily due to the use of different IHC staining methods and scoring criteria, and to the fact that neither IHC nor FISH can accurately quantify the abnormal expression of HER2 protein in tumor cells.

This retrospective study on 118 locally advanced or metastatic gastric cancer samples found that IHC scores of 0/1+ negative (20 cases) and 3+ positive (53 cases) correlated well with FISH detection results, but there is a low FISH positive rate among the IHC 2+ cases, accounting for 38.1% of the total sample size. Among these 45 samples, 24 cases were negative by FISH (53.3%), and only 21 cases were FISH positive (46.7%). Using HER2-SRM detection, the number of samples in the range of 300-700 amol/µg was 23 (19.5% of the total sample size), which was approximately half of the number of IHC 2+ cases (23 vs 45). We also found that regardless of the 45 cases of IHC or the 23 cases of HER2-SRM samples, about half of the samples were HER2-FISH positive (IHC accounted for 46.7%; SRM accounted for 52.2%), and the other half were FISH negative (IHC accounted for 53.3%; SRM accounted for 47.8%). The scatter plot in **Figure 6B** shows the relationship between HER2-SRM and HER2/CEP17 ratio (FISH) and it is shown that in the range of 300-700 amol/µg, both FISH-negative and positive samples are relatively evenly distributed in the x-axis direction, and the projections of the two on the x-axis overlap completely. On the y-axis, it can be seen that some FISH results have samples with high HER2 expression, but the expression detected by HER2-SRM is relatively low. The experiment shows that the direct and accurate quantification of HER2 expression in tumor cells at the amol/µg level is the advantage of the SRM method. Since IHC cannot accurately quantify, we estimate that this phenomenon should be similar to the situation on the x-axis of SRM. If samples detected by SRM in the range of 300-700 amol/µg and samples that cannot be directly judged by IHC are excluded, then the sensitivity of HER2-IHC is 68%, and the specificity is 39%, whereas the sensitivity of HER2-SRM is 59%, and the specificity is 75%. The difference in sensitivity between the two methods is not significant, but the specificity of MS-SRM is significantly higher than that of IHC.

As shown in **Figure 8**, among the 118 study samples analyzed, the higher the HER2-SRM expression, the higher the rate of IHC positivity (IHC3+), and similarly, the higher the HER2-SRM expression, the greater the HER2/CEP17 ratio in FISH detection. Overall, the judgment direction of HER2-IHC, FISH, and HER2-SRM is consistent, but there are a total of 17 samples with low HER2-SRM expression and a high HER2/CEP17 ratio. The samples with HER2-SRM expression less than 300 amol/µg but with a HER2/CEP17 ratio greater than 2 comprised 14.4% of the total sample size and included 8 cases of intestinal type, 3 cases of mixed type, 4 cases of diffuse type, and 2 cases of undifferentiated type. The difference in judgment between HER2-SRM and FISH for these 17 samples is similar to the views of other researchers and requires further subtype research (31).

We also validated the accuracy of the HER2-negative and positive detection limits (300-700 amol/µg) in MS-SRM. One hundred and eighteen samples were included in the study, with two sets of 59 samples collected at different time points. One set was used as an exploration set and the other as a validation set. After analyzing the data from the exploration and validation sets, we found that the sensitivity and specificity of the HER2-negative and positive determination were 61% and 74%, respectively, which were similar to those obtained from the 118 samples. Therefore, the determination limits of HER2-negative and positive in the HER2-SRM method (300-700 amol/µg) were found to have good accuracy and stability.

A study conducted in the United States tested 139 gastroesophageal cancer FFPE samples for HER2 detection using MS-SRM. They also established an upper threshold of 750 amol/µg and a lower threshold of 450 amol/µg, both of which were higher than the upper and lower thresholds in this study. They achieved 100% specificity at the upper threshold and 75% sensitivity at the lower threshold, whereas in this study, 100% specificity was achieved at the upper threshold and 76.7% sensitivity at the lower threshold (43). From the perspective of the upper and lower thresholds, the difference between the two studies was not statistically significant.

Currently, the detection of dynamic targeted proteins is generally considered to be closer to clinical phenotype than relatively static gene testing. Over the past decade, people have increasingly recognized that many seemingly identical tumor patients have different responses to the same treatment, and have also realized that no two cancer patients have exactly the same cancer. Therefore, each cancer patient may respond differently to the same treatments such as chemotherapy, radiation therapy, or targeted therapy (5). The establishment of this method is helpful for exploring the different protein changes and molecular phenotypes of tumors, studying the molecular properties of individual patient tumors, objectively formulating overall clinical treatment plans for patients, and evaluating which populations may benefit from specific clinical treatments and interventions. Moreover, this method does not rely on IHC pathological diagnosis and can quantitatively evaluate the target proteins in tumor cells independently and specifically.

For gastric cancer, patients with negative or low HER2 expression detected by HER2-SRM method may have poor response to anti-HER2 targeted drugs and should not receive anti-HER2 targeted therapy. However, further clinical verification is needed.

We also conducted a study comparing HER2-SRM with FISH in gastric adenocarcinoma biopsy samples. The diagnosis of gastric cancer mainly relies on endoscopic examination and biopsy. In some Chinese gastric cancer patients, the disease is already in advanced stage at the initial diagnosis, and some patients have lost the conditions for surgical resection and only have live tissue examination for evaluating HER2 expression status. Gastric cancer biopsy samples are different from conventional surgical samples in that they are small in volume, and it is necessary to confirm whether FFPE can meet the requirements of targeted protein detection and achieve accurate target protein quantification for clinical application. Among 22 gastric adenocarcinoma biopsy samples, one sample had FISH negative and SRM detection value less than 300 amol/µg, while 11 samples had FISH positive (IHC3+) and SRM detection value greater than 700 amol/µg. The research results showed that the consistency rate between SRM and FISH detection results was 54.55%. The preliminary results of the study are similar to the views of some researchers (33), and also indicate that the established SRM method has good precision and accuracy, and can achieve absolute quantification of HER2 in small samples.

The expression rate of HER2 is associated with intestinal and well-differentiated gastric cancer. Some researchers believe that intestinal type gastric cancer is more likely to occur in the proximal part and that different pathogenic factors may play a role in cancer initiation in these two special anatomical sites and microenvironments (44). The established SRM method was used to preliminarily study the accurate classification (subgrouping) of intestinal and non-intestinal types in patients with gastric adenocarcinoma mixed type (Lauren classification).

Lauren classification was first proposed in 1965, and Lauren divided gastric cancer into intestinal type, diffuse type, mixed type, and uncertain/unclassified type based on different epidemiological and clinical pathological characteristics (45). In 1995, Carneiro et al. conducted a study on 213 gastric cancer patients who were potentially curable by surgery, and based on the original Lauren classification, they improved it by classifying “unclassified” cancers into solid and mixed cancers (46). The revised Lauren classification is divided into intestinal type, diffuse type, mixed type, and solid type (solid cancer), and the proportion of each component in mixed type is defined as ≥5%. It was also found that the biological behavior of mixed type gastric cancer is indeed very different from that of cancers composed of a single morphological component, and its patient survival rate is significantly lower than the other three subtypes. In 2010, WHO classified gastric cancer into papillary type, tubular type, mucinous type, diffuse type (including signet ring cells), mixed type, and other rare types. Considering the degree of tumor differentiation, papillary and tubular types were further divided into high, medium, and low-grade adenocarcinomas (47). The revised Lauren classification and WHO classification have their own characteristics, both of which propose the concept of mixed type gastric cancer, but there is still no unified definition for the proportion of each component in mixed type gastric cancer. The confusion in the definition of mixed type gastric cancer has brought difficulties to the deepening study and individualized clinical treatment of this part of gastric cancer. Subsequently, some Asian scholars’ studies have also obtained similar results (48, 49). Currently, there are still certain limitations in the Cancer Genome Atlas (TCGA) and Asian Cancer Research Group (ACRG) classifications for clearer stratification and selection of patients, which have limited guidance for clinical drug treatment (50, 51).

The study focused on 27 patients with mixed-type gastric adenocarcinoma (Lauren classification) expressing HER2 (IHC 1+, 2+, 3+). A linear mathematical model was constructed using the HER2-SRM expression levels detected in these 27 patients to predict whether mixed-type gastric adenocarcinoma had intestinal phenotype features by ROC curve analysis (as shown in **Figure 9B**). The expression level was found to have a high discriminatory power for identifying the intestinal phenotype of this type, with an AUC of 0.864. Based on the trade-offs between sensitivity and specificity under different criteria, a specific threshold of 700 amol/µg of HER2 protein expression was found to have 87.5% specificity and 81.8% sensitivity. Only 4 out of 27 patients could not be predicted, indicating good accuracy of this method for determining whether mixed-type gastric adenocarcinoma has intestinal phenotype features. Therefore, we defined the expression level of 700 amol/µg as an important indicator for distinguishing between intestinal and non-intestinal types of mixed-type gastric adenocarcinoma and as a reference threshold for the potential benefit of anti-HER2 targeted therapy in this type of patient. Based on these findings, selecting HER2-positive patients from this type of patient for anti-HER2 targeted therapy may benefit some patients, but further clinical validation is needed.

In summary, the advantages of the MS-SRM method are significant and do not need to be elaborated on. However, to achieve absolute quantification of target proteins within tumor cells at the level of 10^-15^, the mass spectrometer requires high sensitivity, specificity, stability, and throughput, as well as a good laser cell cutting system. Therefore, the threshold for this detection platform is relatively high. However, the significant difference between MS-SRM and IHC (antigen-antibody method) is that one injection of MS-SRM can quantify tens, hundreds, or even more target proteins, which reduces the cost of detecting a single target protein. High throughput MS-SRM can objectively guide drug use and evaluate prognosis in cancer patients. It can also be used as a method for screening of potential beneficiary groups of tumors patients, and can serve as a companion diagnostic method for the development of cancer new drugs. It is complementary to methods such as IHC and FISH, and its advantages are even more significant. We also hope that high-end instrument developers can develop small-scale models, small target protein panels and simple data analysis software, these can be suitable for clinical hospitals and general medical laboratories while ensuring high instrument performance. Additionally, objectively speaking, there is a certain gap between the hardware and software of medical research laboratories or medical laboratories in developing countries compared to the United States. Therefore, it is necessary to combine the characteristics and conditions of their own laboratories and introduce and create a suitable experimental platform for themselves to carry out relevant clinical and basic research. Our work has a certain demonstration effect.

While the authors have strived to impartially interpret the results, we acknowledge the possibility of biases in the analysis. These biases could stem from various sources such as data collection procedures, data analysis, or the interpretation of the results. Our efforts to mitigate these biases notwithstanding, readers should exercise discretion when interpreting our findings. It is important to approach our results with a critical eye and to take into account the potential limitations of our analysis.

## Conclusion

Using the MS-SRM method established, 118 FFPE samples from patients in northern China with advanced gastric adenocarcinoma were evaluated for HER2 expression, and the results were compared to the guideline IHC and FISH methods. This study demonstrated that the MS-SRM method can overcome the limitations and deficiencies of IHC, directly quantify the expression of HER2 protein in tumor cells, and be used as a supplement to IHC. It has the potential to be used as a companion diagnosis for new drugs used to treat advanced gastric cancer. Large-scale clinical validation is required.

We established and validated the upper and lower detection limits for abnormal HER2 protein expression in advanced gastric cancer (300-700 amol/µg). This may benefit patients with positive HER2 expression receiving targeted therapy for advanced gastric cancer and has clinical application value. A preliminary clinical study was conducted on the accurate classification (subtyping) of intestinal and non-intestinal types in patients with mixed gastric adenocarcinoma (Lauren classification) and it was proposed that the expression level of HER2 protein at 700 amol/µg could be an important indicator for the classification of intestinal and non-intestinal types in this type of patients, which may potentially benefit patients with intestinal type in HER2-targeted therapy.

Although this study has yielded significant experimental data, there are still limitations. First, the sample size of the study on advanced gastric cancer is small, particularly the study samples of gastric cancer biopsies, intestinal-type gastric cancer, and non-intestinal-type gastric cancer, which may introduce bias into the statistical analysis of the data. Second, there may be subjective interpretation bias in IHC and FISH. Finally, this project is a retrospective study. and important clinical information data, such as OS and PFS, were missing, preventing a good clinical statistical analysis and validation. These issues need to be addressed in future studies.

## Data availability statement

The data that support the findings of this study are openly available in jPOSTrepo at https://repository.jpostdb.org/entry/JPST002128, accession number PXD041613.

## Ethics statement

Written informed consent was obtained from the individual(s) for the publication of any potentially identifiable images or data included in this article.

## Author contributions

BX: Conceptualization, writing-original draft, resources. HC: Conceptualization, methodology. JZ: Formal analysis, investigation, resources, data curation, writing-original draft, supervision, visualization. YC: Formal analysis, data curation, writing-original draft, visualization. LN: Methodology. LC: Conceptualization, writing-review and editing. YuZ: Investigation, resources. YoZ: Conceptualization. ZS: Supervision. YM: Resources, formal analysis. WLL: Writing-review and editing. LH: Methodology. YL: Conceptualization, methodology, supervision. FZ: Conceptualization, methodology, validation, writing-original draft, writing-review and editing, supervision. All authors contributed to the article and approved the submitted version.
