# Supplementary material for: A comparative study of gastric adenocarcinoma HER2 IHC phenotype and mass spectrometry-based quantification
Source: Front Oncol. 2023 Jun 7;13:1152895. doi: 10.3389/fonc.2023.1152895 (PMC10283037; doi:10.3389/fonc.2023.1152895)
Supplement: Supplementary file 1 [file DataSheet_1.docx]

**Supplementary tables**

Table S1. Comparison table of advantages and disadvantages of IHC, FISH and MS-SRM

| detection method | detection principle | advantage | disadvantage |
| --- | --- | --- | --- |
| IHC | based on the principle of antigen-antibody binding, through chemical reaction, using antibody chromogen to develop color, detect antigen in tissue cells | traditional method,  low cost | semi-quantitative, with large differences in subjective judgments |
| FISH | use fluorescently labeled nucleic acids of known sequence as probes to hybridize with nucleic acids in cells or tissue sections to achieve precise positioning of nucleic acid sequences | gold standard,  high sensitivity,  good specificity, | complicated steps and the high cost,  qualitative detection, not quantitative |
| MS-SRM | isotope-labeled internal standard peptide for objective quantification | high sensitivity,  good specificity,  high throughput,  absolute quantification | high technical requirement |

Table S2. Clinicopathological features of 118 gastric adenocarcinoma specimens

| feature | class | count |
| --- | --- | --- |
| age | <63 | 54 |
|  | >=63 | 64 |
| gender | male | 87 |
|  | female | 31 |
| part | fundus-cardia | 15 |
|  | gastric body | 19 |
|  | gastric antrum | 84 |
| WHO classification | moderately-well differentiated adenocarcinoma | 86 |
|  | poorly differentiated adenocarcinoma | 32 |
| Lauren classification | intestinal and mixed | 90 |
|  | diffuse | 28 |
| IHC | 0 or 1+ | 20 |
|  | 2+ | 45 |
|  | 3+ | 53 |
| FISH | negative | 45 |
|  | positive | 73 |

Table S3. Reproducible experimental results of three different types of cancers at different time points

| sample serial number | cancer type | [HER2] content (amol/µg) | |
| --- | --- | --- | --- |
|  |  | freshly prepared tumor tissue sections | preserved for 12 months |
| BD0401C1 | colon cancer | 440 | 384 |
| BD0401C3 | colon cancer | ND | ND |
| BF1103C2 | breast cancer | 427 | 482 |
| BF1103C3 | breast cancer | 634 | 679 |
| BR1006C1 | lung cancer | ND | ND |
| BR1006C3 | lung cancer | ND | ND |
| REF0026 | lung cancer | 13119 | 13956 |
| REF0069 | breast cancer | 344 | 479 |
| REF0008 | breast cancer | 278 | 264 |

Note: ND means that the detection value of mass spectrometry is less than LOQ

Table S4. Statistical table of baseline data of research objects

|  | FISH  positive | FISH  negative | Statistical Inference Test Statistics | p-value |
| --- | --- | --- | --- | --- |
| Grouping | 73 | 45 |  |  |
| IHC, median (IQR) | 3 (2,3) | 2 (1,2) | Ranksum test | <0.001 |
| HER2 CEP 17, median (IQR) | 7.7 (4.4,12.7) | 1.2  (1.1,1.5) | Ranksum test | <0.001 |
| HER2 SRM, median (IQR) | 1031.2 (352,3121.2) | 0  (0,0) | Ranksum test | <0.001 |
| Gender, N (%) |  |  | Chi-sq. (1 df) = 0.62 | 0.433 |
| male | 52 (71.2) | 35 (77.8) |  |  |
| female | 21 (28.8) | 10 (22.2) |  |  |
| Age, median (IQR) | 61.9 (11.1) | 64.7 (9.1) | t-test (116 df) = 1.4 | 0.166 |
| Lauren classification, N (%) |  |  | Chi-sq. (2 df) = 1.99 | 0.37 |
| intestinal type | 42 (57.5) | 20 (44.4) |  |  |
| Hybrid | 16 (21.9) | 12 (26.7) |  |  |
| Diffuse | 15 (20.5) | 13 (28.9) |  |  |
| Lymph node metastasis, N (%) |  |  | Chi-sq. (1 df) = 4.44 | 0.035 |
| no | 10 (19.2) | 17 (38.6) |  |  |
| yes | 42 (80.8) | 27 (61.4) |  |  |
| Degree of differentiation, N (%) |  |  | Chi-sq. (2 df) = 4.19 | 0.123 |
| Low | 16 (21.9) | 16 (35.6) |  |  |
| high | 6 (8.2) | 6 (13.3) |  |  |
| middle | 51 (69.9) | 23 (51.1) |  |  |
| TNM staging, median (IQR) | 3 (2,3) | 3 (2,3) | Ranksum test | 0.361 |
| Tumor site, N (%) |  |  | Chi-sq. (2 df) = 2.58 | 0.275 |
| Fundus-cardia | 11 (15.1) | 4 (8.9) |  |  |
| gastric antrum | 53 (72.6) | 31 (68.9) |  |  |
| body of stomach | 9 (12.3) | 10 (22.2) |  |  |

Note: For TNM staging and lymph node metastasis, a total of 96 cases underwent surgery samples, and the remaining 22 cases were biopsy samples (without TNM staging and lymph node metastasis statistics).

Table S5. Statistical table of baseline data of exploration set and verification set

|  | Explore set | Validation set | Statistical Inference Test Statistics | p-value |
| --- | --- | --- | --- | --- |
| Grouping | 59 | 59 |  |  |
| IHC, median (IQR) | 2 (2,3) | 2 (2,3) | Ranksum test | 0.757 |
| FISH, N (%) |  |  | Chi-sq. (1 df) = 0.04 | 0.85 |
| Neg | 23 (39) | 22 (37.3) |  |  |
| Pos | 36 (61) | 37 (62.7) |  |  |
| HER2 CEP17, median (IQR) | 4 (1.3,7.4) | 2.7 (1.5,9.7) | Ranksum test | 0.6 |
| HER2 SRM, median (IQR) | 448.2 (0,1490.9) | 349.1 (0,1382.7) | Ranksum test | 0.775 |
| Gender, N (%) |  |  | Chi-sq. (1 df) = 0.04 | 0.834 |
| male | 43 (72.9) | 44 (74.6) |  |  |
| female | 16 (27.1) | 15 (25.4) |  |  |
| age, median (IQR) | 63 (56,69) | 65 (59,71) | Ranksum test | 0.175 |
| Lauren classification, N (%) |  |  | Chisq. (2 df) = 3.64 | 0.162 |
| intestinal type | 30 (50.8) | 32 (54.2) |  |  |
| Hybrid | 11 (18.6) | 17 (28.8) |  |  |
| Diffuse | 18 (30.5) | 10 (16.9) |  |  |
| Lymph node metastasis, N (%) |  |  | Chi-sq. (1 df) = 0.91 | 0.339 |
| no | 10 (23.3) | 17 (32.1) |  |  |
| yes | 33 (76.7) | 36 (67.9) |  |  |
| Degree of differentiation, N (%) |  |  | Chi-sq . (2 df) = 5.4 | 0.067 |
| Low | 21 (35.6) | 11 (18.6) |  |  |
| high | 7 (11.9) | 5 (8.5) |  |  |
| middle | 31 (52.5) | 43 (72.9) |  |  |
| TNM staging, median (IQR) | 3 (2,3) | 3 (2,3) | Ranksum test | 0.208 |
| Tumor site, N (%) |  |  | Chi-sq. (2 df) = 0.59 | 0.745 |
| Fundus-cardia | 8 (13.6) | 7 (11.9) |  |  |
| gastric antrum | 43 (72.9) | 41 (69.5) |  |  |
| body of stomach | 8 (13.6) | 11 (18.6) |  |  |

Note: For TNM staging and lymph node metastasis, a total of 96 cases underwent surgery samples, and the remaining 22 cases were biopsy samples (without TNM staging and lymph node metastasis statistics).

Table S6. Basic clinical conditions and tumor characteristics of 22 patients with gastric adenocarcinoma biopsy samples

| feature | class | count |
| --- | --- | --- |
| age | <60 | 11 |
|  | >=60 | 11 |
| gender | male | 16 |
|  | female | 6 |
| part | gastric body | 10 |
|  | gastric antrum | 12 |
| WHO classification | moderately-well differentiated adenocarcinoma | 14 |
|  | poorly differentiated adenocarcinoma | 8 |
| Lauren classification | intestinal and mixed | 14 |
|  | diffuse | 8 |
| IHC | 2+ | 19 |
|  | 3+ | 1 |
| FISH | negative | 1 |
|  | positive | 21 |

Table S7. Statistics of HER2-IHC, FISH and SRM results in 22 cases of gastric adenocarcinoma biopsy samples

| group | SRM < 300 amol/µg | 300-700  amol/µg | SRM > 700 amol/µg |
| --- | --- | --- | --- |
| IHC2+ | 2 | 1 | 0 |
| IHC3+ | 2 | 6 | 11 |
| FISH (-) | 1 | 0 | 0 |
| FISH (+) | 3 | 7 | 11 |

Table S8. Clinicopathological features of 27 gastric adenocarcinoma mixed type (Lauren type) samples

| feature | class | count |
| --- | --- | --- |
| age | <63 | 11 |
|  | >=63 | 16 |
| gender | male | 18 |
|  | female | 9 |
| part | gastric body | 6 |
|  | gastric antrum | 21 |
| WHO classification | tubular adenocarcinoma | 5 |
|  | hepatic adenocarcinoma | 1 |
|  | poorly differentiated adenocarcinoma | 21 |
| lymphatic metastasis | yes | 22 |
|  | no | 5 |
| IHC | 0 or 1+ | 5 |
|  | 2+ | 12 |
|  | 3+ | 10 |
| FISH | negative | 12 |
|  | positive | 15 |

Table S9. HER2-SRM interpretation has enterotype phenotype characteristics pathologically confusion matrix

| confusion matrix | | HER2-SRM interpretation | | total |
| --- | --- | --- | --- | --- |
|  |  | benefit (may) | benefit (not) |  |
| pathological interpretation | enterotype phenotype (yes) | 9 | 2 | 11 |
|  | enterotype phenotype (none) | 2 | 14 | 16 |
| total | | 11 | 16 | 27 |
